# Characteristics of Small Intestinal Diseases on Single-Balloon Enteroscopy: A Single-Center Study Conducted Over 6 Years in China

**DOI:** 10.1097/MD.0000000000001652

**Published:** 2015-10-23

**Authors:** Zhang Tao, G.X. Liu, L. Cai, H. Yu, X.J. Min, H.T. Gan, K. Yang, Li SQ, J. Yan, L. Chen, Q.H. Tan, J.C. Wu, X.L. Huang

**Affiliations:** From the Gastroenterology, West China Hospital, Sichuan University, Gastroenterology, Nanchong Central Hospital (ZT); Gastroenterology and Geriatrics, West China Hospital, Sichuan University (LGX, YK, YJ); Gastroenterology, West China Hospital, Sichuan University (CL, YH, LC, QHT, JCW, XLH); Endoscopy Center, West China Hospital, Sichuan University (MXJ); and Gastroenterology, Nanchong Central Hospital (LSQ).

## Abstract

The small intestine has been considered inaccessible for a long term. The development of single-balloon endoscopy has greatly improved the diagnosis and treatment possibilities for small intestinal diseases.

In this study, we aimed to explore the demographic characteristics and small intestinal diseases of patients who underwent single-balloon enteroscopy between 2009 and 2014 at our endoscopy center. We determined the enteroscopic findings for each small intestinal disease and the most susceptible age groups.

In total, 186 patients were included in the study. Their mean age was 45.87 ± 15.77 years. Patients who underwent single-balloon enteroscopy were found to have neoplasms (most common age group: 14–45 years, most common lesion location: jejunum), lymphoma (46–59 and 60–74 years, ileum), protuberant lesions (45–59 years, jejunum), inflammation (14–45 and 46–59 years, ileum), benign ulcers (14–45 years, jejunum), diverticulum (14–45 years, ileum), vascular malformations (60–74 years, jejunum), polyps (14–45 years, jejunum), Crohn's disease (14–45 years, jejunum), hookworm infection (14–45 years, jejunum), lipid pigmentation (14–45 and 46–59 years, jejunum), undetermined bleeding (46–59 years, ileum), or undetermined stenosis (31 years, duodenum). Each small intestinal disease had distinct enteroscopic findings.

## INTRODUCTION

The small intestine has been considered inaccessible for a long time because of its long length.^1^ The advent of capsule endoscopy and double-balloon or single-balloon endoscopy has enabled the exploration of the small bowel thus facilitating the detection of small intestinal diseases. Specifically, double-balloon or single-balloon endoscopy has facilitated biopsy and therapeutic interventions for small intestinal diseases,^2^ which have greatly improved the diagnosis and treatment of small intestinal diseases.^3^ The SBE characteristics of small intestinal diseases have not be reported recently. For enteroscopists, after they found a lesion under SBE, there is no reference for them to consider the possible small intestinal disease. For clinicians, they cannot deduce the possible disease for a patient with a possible small intestinal disease from the age group and symptoms. To explore the characteristics of small intestinal diseases, we retrospectively analyzed the results of single-balloon enteroscopy carried out at our institute.

## METHODS

This retrospective study was conducted at the endoscopy center of our academic hospital between January 2009 and December 2014. The study was approved by the Ethics Committee of Sichuan University. Single-balloon enteroscopy was performed by experienced endoscopists in all patients. Patient demographic and clinical data such as indications for enteroscopy, enteroscopy approach (antegrade, retrograde, or antegrade and retrograde), endoscopic diagnosis, and images were collected. For patients who underwent a biopsy under enteroscopic guidance, pathological findings were also collected.

Single-balloon enteroscopy was performed for obscure (unknown origin after gastroscopy and colonoscopy), gastrointestinal bleeding (overt or occult bleeding),^4^ unexplained abdominal pain and diarrhea, and suspected tumors in the small bowel. Patients were classified into 5 different age groups^5^—<14 years old, 14 to 45 years old, 46 to 59 years old, 60 to 74 years old, 75 to 89 years old, and >90 years old.^7^ Single-balloon enteroscopy was performed in patients with contraindications for gastrointestinal endoscopy, acute pancreatitis, acute biliary tract infection, severe esophageal injury, or extensive abdominal adhesion. All patients provided informed consent for the performance of single-balloon enteroscopy.

In this study, we determined the indications for single-balloon enteroscopy and compared these according to the chosen procedure approach, by using the chi-square test. The nature of the different small intestinal diseases was elucidated, and finally, we examined the demographic and clinical characteristics for each diagnosis, such as mean patient age, male-to-female ratio, susceptible age group, and susceptible location within the small intestine, as well as the enteroscopic characteristics.

## RESULTS

### Demographic and Clinical Characteristics of the Study Population

In total, 186 patients underwent 196 procedures. The mean patient age was 45.87 ± 15.77 years, and the mode was 46 years. The male-to-female and antegrade-to-retrograde single-balloon enteroscopy ratios were 1.2:1 and 1.5:1, respectively. The indications for single-balloon enteroscopy are shown in Table 1. These included overt bleeding, occult bleeding, and suspected tumor and did not differ according to the single-balloon enteroscopy approach used (antegrade or retrograde). Thus, the approach seemed to have been chosen not according to the indication, but perhaps depending on the lesion. However, for diarrhea, antegrade single-balloon enteroscopy was always chosen.

**TABLE 1:**
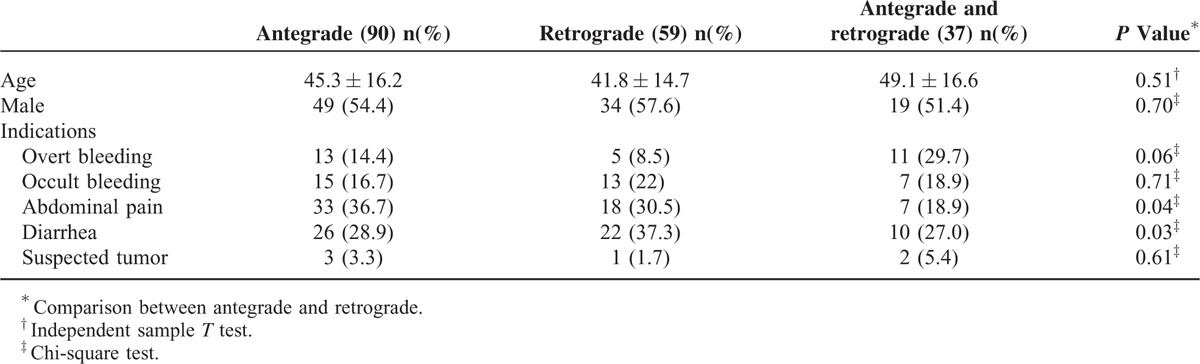
General Data of Included Patients

### Single-Balloon Enteroscopic Findings

Of the 186 patients, 57 had normal findings and 129 had abnormal findings on single-balloon enteroscopy (Table 2). Among the abnormal patients, 85 underwent a biopsy or were treated. Of the patients with abnormal findings, 94 had benign disease, 27 had malignant disease, and 8 had an undetermined condition. The most common malignant disease was neoplasm, followed by lymphoma and malignant protuberant lesions. The most common benign condition was inflammation, followed by benign ulcers, diverticulum, protuberant lesions, vascular malformations, polyps, Crohn's disease, hookworm infection, and lipid pigmentation (observation of a few white or yellow particles confirmed to be lipid by the pathological examination). For undetermined conditions, undetermined bleeding was the most common, followed by stenosis.

**TABLE 2:**
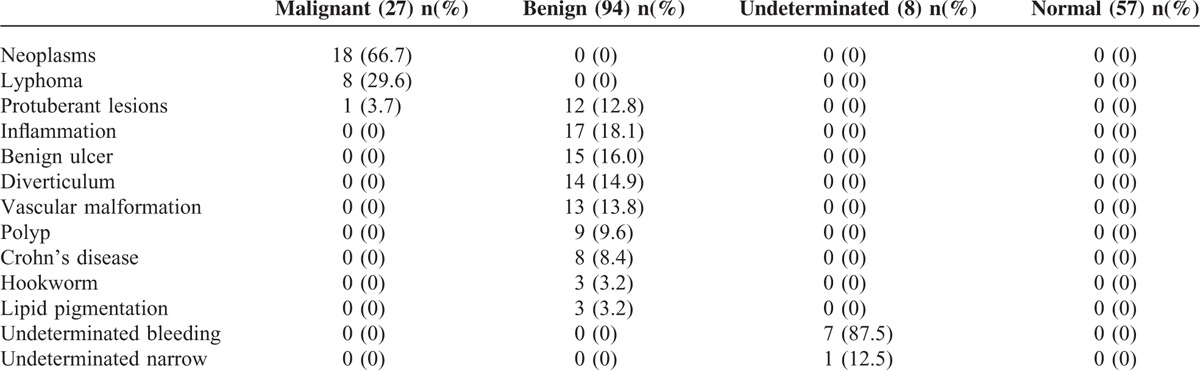
Nature of Enteroscopic Lesions

The procedure approach and the depth of insertion for the different small intestinal diseases are shown in Table 3. Both antegrade and retrograde procedures were performed in only 37 patients, only 7 of whom had normal findings. The depth of insertion with antegrade and retrograde procedures were 200.50 ± 61.57 and 124.07 ± 59.30 cm, respectively. The characteristics of the individual small intestinal lesions are show in Table 4.

**TABLE 3:**
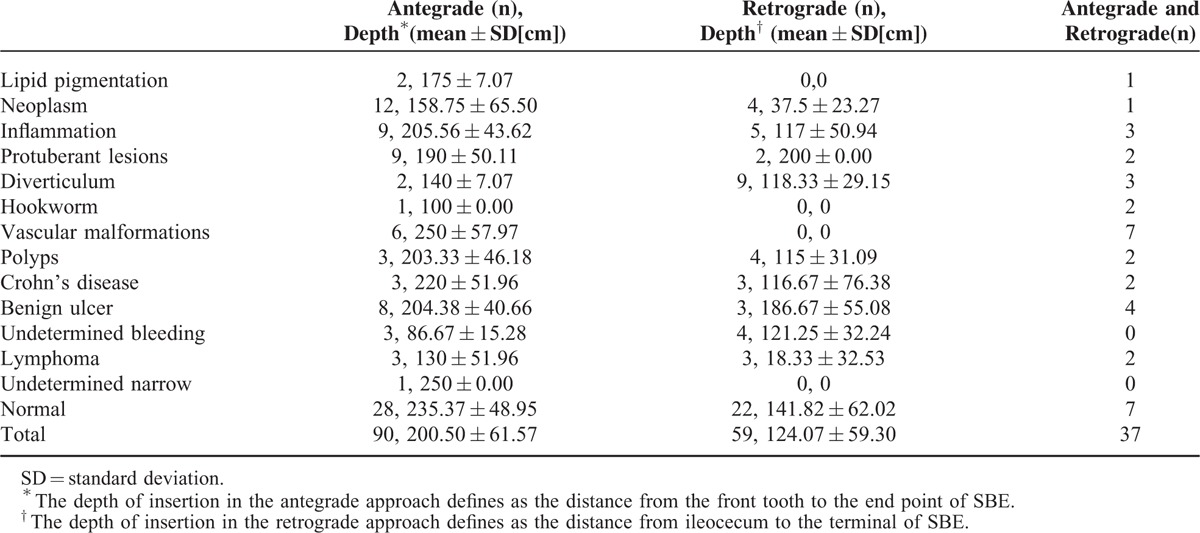
Procedure Approaches and Depths of Insertion in Different Small Intestinal Diseases

**TABLE 4:**
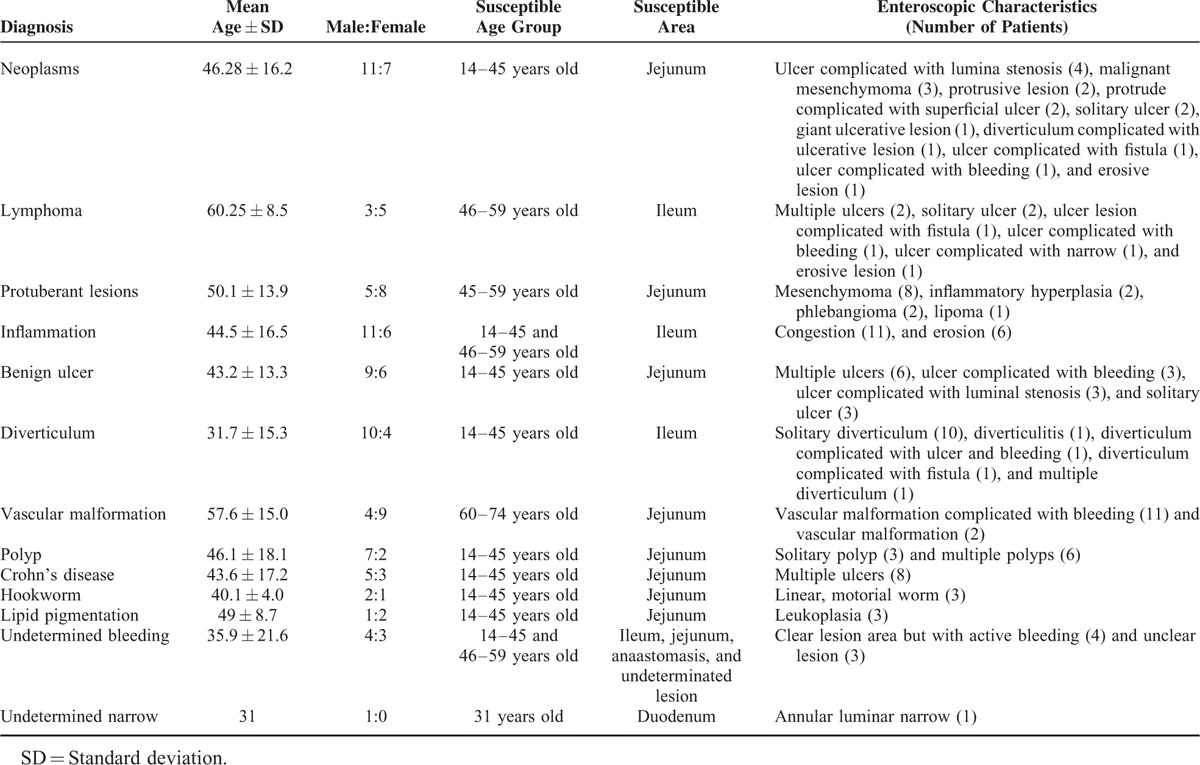
Characteristics of Small Intestinal Lesions

### Neoplasms

**FIGURE 1:**
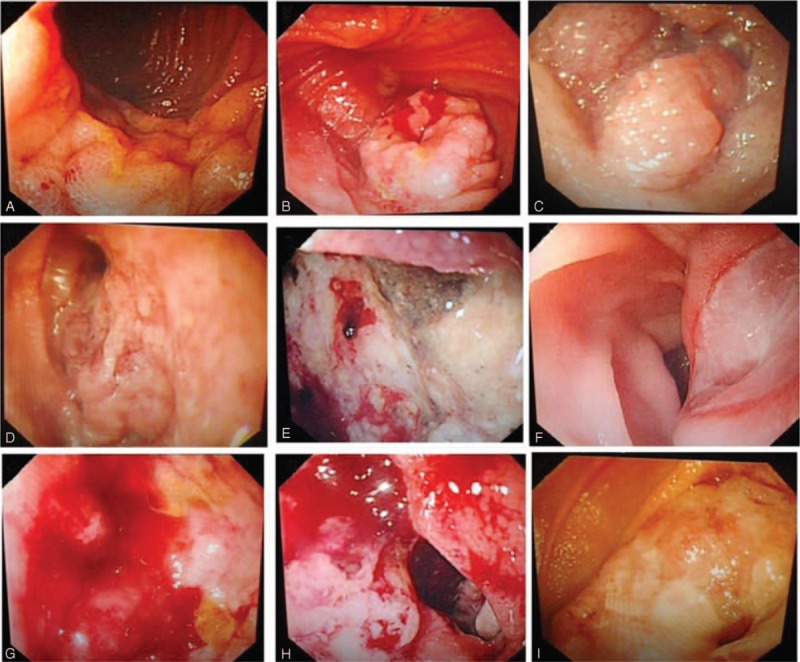
Neoplasms. A, Ileum erosive and coarse lesion; B, ileum malignant mesenchymoma; C, ileum protrusive lesion; D, ileum solitary ulcer; E, jejunum giant ulcerative lesion; F, jejunum protrusive lesion complicated with superficial ulcer; G, jejunum ulcer complicated with bleeding; H, jejunum ulcerative lesion; I, jejunum ulcer complicated with luminar stenosis.

Neoplasms were found in 18 patients. Their mean age was 46.28 ± 16.2 years, and the male-to-female ratio was 1.6:1. Patients in the 14 to 45 years age group were the most susceptible, and the jejunum was the most common lesion location. On enteroscopy, the neoplasms were seen as ulcers complicated with luminal stenosis (n = 4), malignant mesenchymoma (n = 3), protrusive lesions (n = 2), protrusive lesions complicated with superficial ulcers (n = 2), solitary ulcers (n = 2), giant ulcerative lesions (n = 1), diverticulum complicated with ulcerative lesions (n = 1), ulcers complicated with fistula (n = 1), ulcers complicated with bleeding (n = 1), or erosive lesions (n = 1) (Figure 1).

### Lymphoma

**FIGURE 2:**
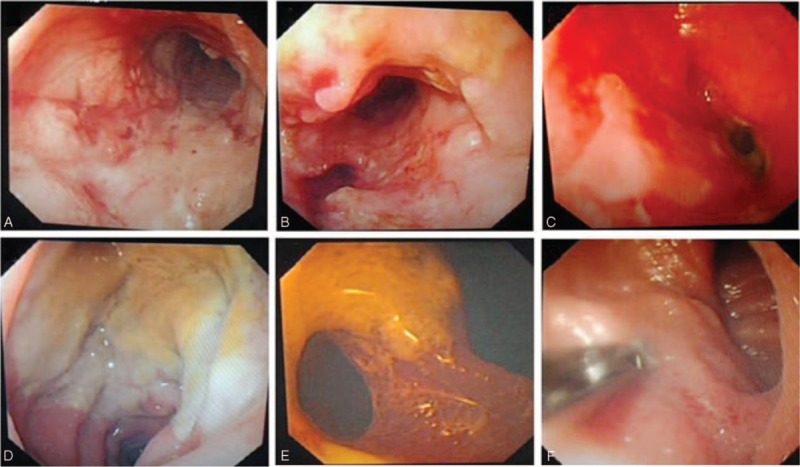
Lymphoma. A, Duodendum ulcer complicated with bleeding; B, ileum ulcer; C, ileum ulcer complicated with bleeding; D, jejunum solitary ulcer; E, ulcer complicated with fistula.

Eight patients were diagnosed lymphoma. Their mean age was 60.25 ± 8.5 years, and the male-to-female ratio was 1:1.7. Patients in the 46 to 59 and 60 to 74 years age groups were the most susceptible, and the ileum was the most common lesion location. On enteroscopy, the lymphomas were seen as multiple ulcers (n = 2, 25%), solitary ulcers (n = 2, 25%), ulcers complicated with fistula (n = 1, 12.5%), ulcers complicated with bleeding (n = 1, 12.5%), ulcers complicated with stenosis (n = 1, 12.5%), or erosive lesions (n = 1, 12.5%) (Figure 2). Of note, the erosive lesion in this case was diagnosed as mucosa-associated lymphoid tissue lymphoma based on biopsy and pathological findings.

### Protuberant Lesions

**FIGURE 3:**
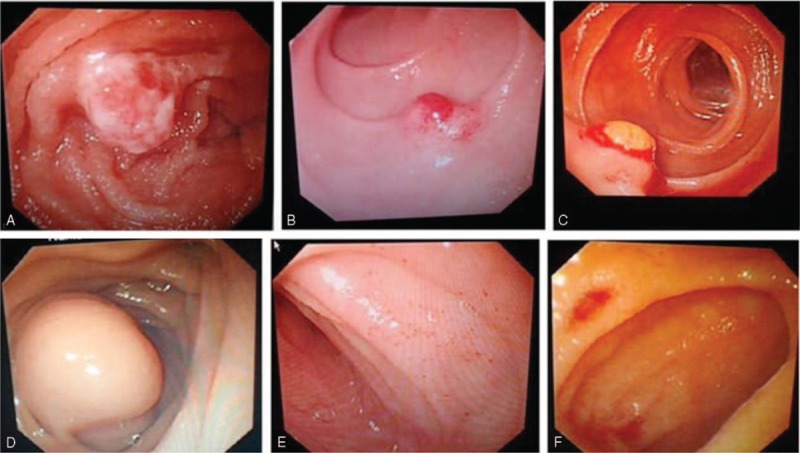
Protuberant lesions and inflammation. A, Ileum angeioma; B, jejunum inflammatory hyperplasia; C, jejunum lipoma; D, jejunum mesenchymoma; E, jejunum congestion; F, ileum erosion.

Fourteen patients had protuberant lesions. “Protuberant” was defined as elevation above the mucosa.^6–8^ The mean patient age was 50.1 ± 13.9 years, and the male-to-female ratio was 1:1.8. Patients in the 45 to 59 years age group were the most susceptible, and the jejunum was the most common lesion location (n = 7), followed by the ileum (n = 3), and duodenum (n = 3). On single-balloon enteroscopy, protuberant lesions were seen as mesenchymoma (n = 8, 61.5%), inflammatory hyperplasia (n = 2, 15.4%), phlebangioma (n = 2, 15.4%), or lipoma (n = 1, 7.7%) (Figure 3). Mesenchymoma was diagnosed by the pathological examination of surgical specimens, and 1 of these lesions was considered to be a neoplasm based on the pathological examination. Inflammatory hyperplasia was diagnosed by biopsy; phlebangioma was mainly diagnosed by gross observation, based on light blue coloration; and lipoma was diagnosed based on biopsy and gross observation.

### Small Intestinal Inflammation

Seventeen patients had small intestinal inflammation. Their mean age was 44.5 ± 16.5 years, and the male-to-female ratio was 1.8:1. Patients in the 14 to 45 and 46 to 59 years age groups were the most susceptible, and the ileum was the most common lesion location (n = 8), followed by the jejunum (n = 5), and duodenum (n = 4). On enteroscopy, small intestinal inflammation was seen as congestion (n = 11, 64.7%) or erosion (n = 6, 35.3%) (Figure 3).

### Benign Ulcer

**FIGURE 4:**
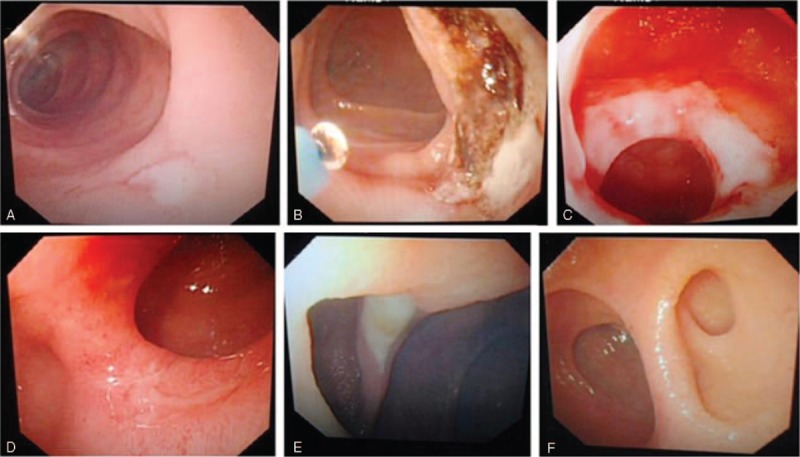
Benign ulcer and diverticulum. A, Ileum ulcer; B, jejunum solitary ulcer complicated with bleeding; C, jejunum ulcer complicated with luminal stenosis; D, ileum diverticulitis; E, ileum diverticulum complicated with ulcer; F, jejunum multiple diverticulum.

Fifteen patients were diagnosed with benign ulcers. Their mean age was 43.2 ± 13.3 years, and the male-to-female ratio was 1.5:1. Patients in the 14 to 45 years age group were the most susceptible, and the jejunum was the most common lesion location (n = 6), followed by the ileum (n = 5), duodenum (n = 1), stomach (n = 1), jejunum and duodenum (n = 1), and anastomosis (n = 1). The mean diameter of the benign ulcers was 0.5 cm (range: 0.2–0.8 cm). On enteroscopy, benign ulcers were seen as multiple ulcers (n = 6, 40%), ulcers complicated with bleeding (n = 3, 20%), ulcers complicated with luminal stenosis (n = 3, 20%), or solitary ulcers (n = 3, 20%) (Figure 4).

### Diverticulum

Fourteen patients were diagnosed with diverticulum. Their mean age was 31.7 ± 15.3 years, and the male-to-female ratio was 2.5:1. Patients in the 14 to 45 years age group were the most susceptible, and the ileum was the most common lesion location (n = 10), followed by the jejunum (n = 4). On enteroscopy, small intestinal diverticulum was seen as solitary diverticulum (n = 10, 71.4%), diverticulitis (n = 1, 7.1%), diverticulum complicated with ulcers and bleeding (n = 1, 7.1%), diverticulum complicated with fistula (n = 1, 7.1%), or multiple diverticulum (n = 1, 7.1%) (Figure 4).

### Vascular Malformation

**FIGURE 5:**
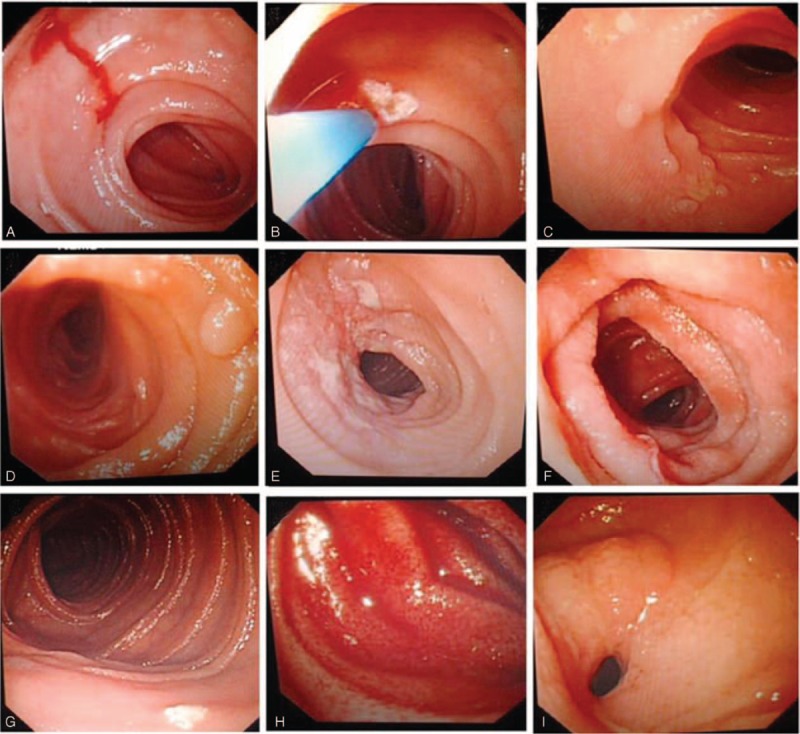
Vascular malformation, polyp, Crohn's disease, Hookworm, lipid pigmentation, and undeterminated narrow and bleeding. A, Preoperative jejunum vascular malformation complicated with bleeding; B, postoperative jejunum vascular malformation complicated with bleeding preoperative postoperative; C, ileum multiple polyps; D, jejunum solitary polyp; E, jejunum multiple ulcers (Crohn's disease); F, jejunum hookworm; G, jejunum leukoplakia; H, undeterminated bleeding; I, undeterminated narrow.

Thirteen patients were diagnosed with vascular malformation. Their mean age was 57.6 ± 15.0 years, and the male-to-female ratio was 1:2.25. Patients in the 60 to 74 years age group were most susceptible, and the jejunum was the most common lesion location (n = 8, 61.5%), followed by the ileum (n = 2, 15.4%), colon and ileum (n = 1, 7.7%), jejunum and ileum (n = 1, 7.7%), and jejunum and duodenum (n = 1, 7.7%). On enteroscopy, vascular malformation was seen as vascular malformation complicated with bleeding (n = 11, 84.6%) or vascular malformation without bleeding (n = 2, 15.4%) (Figure 5).

## POLYP

Nine patients had polyps. Their mean age was 46.1 ± 18.1 years, and the male-to-female ratio was 3.5:1. Patients in the 14 to 45 years age group were the most susceptible, and the jejunum was the most common lesion location (n = 4, 44.4%), followed by the ileum (n = 2, 22.2%), duodenum (n = 2, 22.2%), and colon (n = 1, 11.1%). The mean diameter of the polyps was 0.4 cm (range: 0.2–0.6 cm). On enteroscopy, the polyp were found to be solitary (n = 3, 33.3%) or multiple (n = 6, 66.7%) (Figure 5).

### Crohn's Disease

Eight patients had Crohn's disease. Their mean age was 43.6 ± 17.2 years, and the male-to-female ratio was 1.7:1. Patients in the 14 to 45 years age group were the most susceptible, and the jejunum was the most common lesion location (n = 5), followed by the ileum (n = 3). Crohn's disease was seen as multiple ulcers on enteroscopy in all patients (Figure 5).

### Hookworm Infection

Three patients were diagnosed with hookworm infection. Their mean age was 40.1 ± 4.0 years, and the male-to-female ratio was 2:1. All 3 patients were in the 14 to 45 years age group, and the lesions were located in the jejunum in all of them. Either one or many mobile worms were seen on enteroscopy (Figure 5).

### Lipid Pigmentation

Three patients had lipid pigmentation. Their mean age was 49 ± 8.7 years, and the male-to-female ratio was 1:2. They were in the 14 to 45 or 46 to 59 years age group. Lesions in all 3 were found in the jejunum and were seen as leukoplasia on enteroscopy (Figure 5).

### Undetermined Bleeding

Seven patients had undetermined bleeding. Their mean age was 35.9 ± 21.6 years, and the male-to-female ratio was 4:3. Patients in the 14 to 45 and 46 to 59 years age groups were most susceptible, and lesions were located in the ileum (n = 2), jejunum (n = 1), anastomosis (n = 2), or undetermined areas (n = 2). On enteroscopy, the causative lesions could not be identified because of active bleeding in clear lesions (which could not be biopsied without considerable risk) (n = 4) or unclear lesions (n = 3) (Figure 5).

### Undetermined Stenosis

Only 1 patient had undetermined stenosis; this was a 31-year-old male patient. The lesion was located in the duodenum. Because of the annular luminal stenosis, the depth of insertion of the enteroscope into the small intestine was limited, although the condition of the mucosa was good (Figure 5). Therefore, pathological findings were inconclusive.

## DISCUSSION

Single-balloon enteroscopy enables deep insertion into the small intestine, which not only enables diagnosis as with methods such as capsule endoscopy, but also facilitates lesion biopsy in order to identify the nature of small intestinal lesions and stop bleeding with techniques such as electrocoagulation.

The mean age of patients who underwent enteroscopy in our study was 45.87 years, which is less than that reported in 2 studies in the USA^9–10^ but similar to 2 other studies conducted in developing countries.^11–12^ This could suggest that the age of patients with small intestinal disorders or suspected small intestine disorders differs according to economic levels; however, there is no relevant epidemiological report to support this.

Most of the included patients had benign lesions (73%), and only 21% had malignant diseases, similar to the findings of another study conducted in China.^13^ Eight patients (6%) could not be diagnosed with enteroscopy because of bleeding or luminal stenosis, with most patients in the undetermined conditions group having undetermined bleeding, similar to the findings of a study in the USA.^14^

Neoplasms were located mainly in the jejunum, similar to the case in another Chinese study.^15^ The male-to-female ratio in our study was 1.6:1, similar to the results of another study. However, that study reported a different susceptible age group (∼46 years old).^16^

For small intestinal lymphomas, the male-to-female ratio was 1:1.7 in our study, which is different from the 1.3:1 ratio reported in a previous study, and the mean patient age (60 years old) was higher than previously reported.^17^ Small intestinal lymphomas in our study were noted mainly in the ileum. However, the jejunum and ileum were found to be susceptible locations in a previous Asian study.^18^

The mean age of patients with Crohn's disease was lower than previously reported values^19^

Here, we described the enteroscopic characteristics of different small intestinal diseases—neoplasms, lymphomas, protuberant lesions, small intestinal inflammation, benign ulcers, diverticulum, vascular malformation, polyps, Crohn's disease, hookworm infection, lipid pigmentation, undetermined bleeding, and undetermined stenosis and determined the susceptible age groups. Further studies on the characteristics of small intestinal diseases can provide references for enteroscopists to consider the possible small intestinal disease after finding a lesion under SBE. Moreover, it also can help clinicians to deduce the possible disease for a patient with a possible small intestinal disease from the age group and symptoms.

Some limitations need to be considered. First, the sample size was small. Second, this was a retrospective analysis. All of these cannot ignore selection bias.

Small intestinal diseases examined by single-balloon enteroscopy were most commonly found in young patients (<45 years old) except for lymphoma, protuberant lesions, vascular malformations, and undetermined bleeding. The lesions and enteroscopic characteristics differ for different small intestinal diseases.
